# Application of genetic algorithms and constructive neural networks for the analysis of microarray cancer data

**DOI:** 10.1186/1742-4682-11-S1-S7

**Published:** 2014-05-07

**Authors:** Rafael Marcos Luque-Baena, Daniel Urda, Jose Luis Subirats, Leonardo Franco, Jose M Jerez

**Affiliations:** 1 Department of Computer Science, University of Málaga, Málaga, Spain; 2 Biomedical Research Institute of Málaga (IBIMA), Spain

**Keywords:** Microarray, Genetic algorithms, Constructive neural networks, Feature Selection

## Abstract

**Background:**

Extracting relevant information from microarray data is a very complex task due to the characteristics of the data sets, as they comprise a large number of features while few samples are generally available. In this sense, feature selection is a very important aspect of the analysis helping in the tasks of identifying relevant genes and also for maximizing predictive information.

**Methods:**

Due to its simplicity and speed, Stepwise Forward Selection (SFS) is a widely used feature selection technique. In this work, we carry a comparative study of SFS and Genetic Algorithms (GA) as general frameworks for the analysis of microarray data with the aim of identifying group of genes with high predictive capability and biological relevance. Six standard and machine learning-based techniques (Linear Discriminant Analysis (LDA), Support Vector Machines (SVM), Naive Bayes (NB), C-MANTEC Constructive Neural Network, K-Nearest Neighbors (kNN) and Multilayer perceptron (MLP)) are used within both frameworks using six free-public datasets for the task of predicting cancer outcome.

**Results:**

Better cancer outcome prediction results were obtained using the GA framework noting that this approach, in comparison to the SFS one, leads to a larger selection set, uses a large number of comparison between genetic profiles and thus it is computationally more intensive. Also the GA framework permitted to obtain a set of genes that can be considered to be more biologically relevant. Regarding the different classifiers used standard feedforward neural networks (MLP), LDA and SVM lead to similar and best results, while C-MANTEC and k-NN followed closely but with a lower accuracy. Further, C-MANTEC, MLP and LDA permitted to obtain a more limited set of genes in comparison to SVM, NB and kNN, and in particular C-MANTEC resulted in the most robust classifier in terms of changes in the parameter settings.

**Conclusions:**

This study shows that if prediction accuracy is the objective, the GA-based approach lead to better results respect to the SFS approach, independently of the classifier used. Regarding classifiers, even if C-MANTEC did not achieve the best overall results, the performance was competitive with a very robust behaviour in terms of the parameters of the algorithm, and thus it can be considered as a candidate technique for future studies.

## Introduction

DNA microarray technology has been widely used in cancer studies for prediction of disease outcome [1]. It is a powerful platform successfully used for the analysis of gene expression in a wide variety of experimental studies [2]. However, due to the large number of features (in the order of thousands) and the small number of samples (mostly less than a hundred) in this kind of datasets, microarray data analysis faces the "large-p-small-n" paradigm [3] also known as the curse of dimensionality. In this sense, feature selection preprocessing refers to decide which genes to include in the prediction, and it is a crucial step in developing a class predictor. Including too many features could reduce the model accuracy and may lead to overfit the data [4]. Two different algorithms have been widely used in literature to carry out feature selection, the Stepwise Forward Selection algorithm (SFS) and the Genetic Algorithms (GA). In the SFS algorithm the choice of predictive features is carried out by an automatic procedure that starts from single variable models and tests the addition of each feature using a comparison criterion. This algorithm has been used to identify a predictive gene signature whose size is minimum [5,6]. GA are also well considered as suitable evolutionary strategies for feature selection in problems with a large number of features [7,8], and are applied to different areas, from object detection [9] to gene selection in microarray data [10].

On the other hand, model selection is another important step in the estimation of expression profiles to predict diseases outcome[11]. In this regards, different well-known machine learning-based techniques have been used recently in literature wrapped into features selection algorithms to develop a class predictor, e.g. Support Vector Machines (SVM)[12], Multilayer Perceptron (MLP)[13], k-Nearest Neighborhood (kNN)[14], Linear Discriminant Analysis (LDA)[15] and NaiveBayes. Nevertheless, few of these related works brings together different learning algorithms, features selection schemes and input datasets. Besides, some of them are focused mainly on optimising the prediction accuracy, and lack of any biological analysis for the resulting molecular signatures via specialised software as Ingenuity Pathway Analysis (IPA), GeneOntology (GO) or KEGG [16].

This paper presents an exhaustive analysis of performance for SFS and GA as general frameworks to estimate expression genes profiles from microarray data with high predictive capability and biological relevance. Five standard and machine learning-based techniques (MLP, SVM, kNN, LDA, NaiveBayes) are used within both frameworks using six free-public cancer datasets (breast, colon, leukemia, lung, ovarian and prostate cancer) for the task of predicting cancer outcome. Moreover, an important goal of the present study is to test the performance of a new constructive neural network classification algorithm (C-MANTEC) in the mentioned framework. C-MANTEC have been previously proved to get similar classification results than traditional multi-layer perceptrons (MLP) or support vector machines (SVM), with the advantage that the architecture is dynamically estimated [17]. This is a critical factor in the wrapper selection methods combined with neural networks, because the subsets analysed are different sizes (or even the complexity of the features selected need projections in higher spaces), which implies that the use of the same architecture is not always appropriate. On the other hand, considering that using non redundant variables is commonly preferable in feature selection, the evolutionary strategy presented in this work incorporates a mutual information filter to minimise the correlation between the selected features while increasing the classifier performance. Furthermore, a biological analysis of the relevance of the selected genes is performed using IPA tool, which can lead us to conduct an understanding of microarray data.

## Methodology

Feature selection techniques can be organised into three broad categories: filter, wrapper and embedded methods [18]. Filter methods use statistical properties of the variables to discard poorly descriptive features and are independent of the classifier. Wrapper methods are more computationally demanding than filter methods, as subsets of features are evaluated with a classification algorithm in order to obtain a measure of goodness to be used as the improvement criteria. Embedded methods are also classifier dependent, but they can be viewed as a search in the combined space of feature subsets and classifier models, with the additional restriction that it is not possible to replace the classifier used since feature selection and classification methods work as a whole.

In this work a comparison between a SFS and GA based approach is done. As the data input space is quite large for microarray data a pre-selection approach is first applied in order to reduce the size of the input features to a 5% of the total. After this reduction, six different classifiers are applied within both frameworks.

### Pre-selection step

Since cancer datasets normally contain a large number of genes, a pre-selection step to reduce the initial number of variables is required. In terms of the quality of the features ranked, it has been found that using the Student t-test is generally more successful than other filter methods[19]. In particular, the Welch t-test [20], an adaptation of the t-test, is used for the pre-selection step assuming the two classes (patient has cancer or not) have unknown and unequal variances, as it is not advisable to use the basic t-test if both requirements are not clearly satisfied [18]. A 5% of the total number of genes are retained (between 400 and 2000 genes, approximately, in the datasets selected), which will be the input for the two approaches (SFS and GA) applied, and described below.

### Stepwise forward selection procedure

An exhaustive evaluation of all the possible subsets of *n *features involves a complexity of *O*(2*n*) which becomes infeasible for large values for *n*. In this sense, several heuristic algorithms have been proposed to reduce the computational complexity of wrapper algorithms. Stepwise forward procedures for feature selection analyse the inclusion of one or several features in order to improve the performance of the classification task. Thus, sequential forward selection [21] chooses the best variable in each iteration by minimising the misclassification rate, and includes it in the final subset of features. The algorithm will continue to add variables until the performance stops to improve.

### Evolutionary approach

GAs are a class of optimisation procedure inspired by the biological mechanisms of reproduction. One of the key aspects of GA is the so called fitness function *f*(**x**), that should be maximised or minimised over a given space *X *of arbitrary dimension, in an iterative search process in which the population of selected genes evolves as described in detail below.

#### *Encoding and initial population*

A simple encoding scheme to represent as much as possible of the available information was employed. A string of bits whose length is equal to the total number of genes is used, using a binary variable associated with each bit. If the *i^th ^*bit is active (value 1), then the *i^th ^*gene is selected in the chromosome (a value of 0 indicates that the corresponding feature is ignored). Both, the active features and the number of them were generated randomly, and in all the experiments a population size of 100 individuals was used.

#### *Selection, crossover and mutation*

A selection strategy based on roulette wheel and uniform sampling was applied, while an elite count value of 10 (number of chromosomes which are retained for the next generation) was selected. Scattered crossover, in which each bit of the offspring is chosen randomly, was the choice for combining parents of the previous generation, using a crossover rate set to 0.8. In addition to that, a traditional mutation operator which flips a specific bit with a probability rate of 0.2 was considered. Since it was empirically verified that the best subsets include few features, a modification which involves mutating a random number of bits between 1 and the number of active features of the individual was also applied, as this change avoids the increment on the number of active features in the last generations of the GA.

#### *Fitness function*

The fitness function assesses each chromosome in the population so that it can be ranked against all the other chromosomes. Three aspects where considered for constructing the fitness function: i) The main objective is to obtain the highest performance ii) Among two subsets that achieve equal performance, the one that contains a lower number of features is preferred. iii) The combination of features with low redundancy among them and with a certain resemblance to the target class, are beneficial for improving performance rates [22]. Therefore, the fitness function contains three terms: the misclassification error, the number of features selected and a redundancy measure among them. Datasets are splitted into training and testing sets in order to evaluate the generalisation ability of the proposed chromosome.

Statistical techniques such as mutual information [23] can be used for measuring the correlation between a pair of features. The mutual information between two continuous random variables *y *and *z *is given by the following equation:

(1) $$I\left(y,z\right)=\iint p\left(y,z\right)\text{log}\left(\frac{p\left(y,z\right)}{p\left(y\right)p\left(z\right)}\right)dy\;dz$$

where *p*(*y, z*) is the joint probability density function of *y *and *z*, and *p*(*y*) and *p*(*z*) are the marginal probability density functions of *y *and *z *respectively.

Mutual information is a non-negative quantity, with a zero value indicating that the variables are completely independent. The more correlated two variables are, the greater their mutual information. Advantages of this measure are that the dependency between variables is no longer restricted to linear correlation and that it can handle nominal or discrete features. Although it is hard to compute it for continuous data, the probability densities can be well estimated by discretising it through the use of histograms[24]. A measure which incorporates the correlation of features with the target class and penalises the redundancy among the selected features is described as follows [22]:

(2) $$corr\left(x\right)=\frac{1}{t}\overset{k}{\underset{i=1}{\sum }}\overset{k}{\underset{j=i+1}{\sum }}I\left(x_{j},x_{i}\right)-\frac{1}{k}\overset{k}{\underset{j=1}{\sum }}I\left(x_{j},C\right)$$

where *k *is the number of features selected, *C *is the target class and *t *is the number of combinations between the pairs of the chromosome *x *analysed. Finally, the function to be minimised ( the *f itness*(**x**) function) is represented as follows for a given subset **x**.

(3) $$fitness\left(x\right)=\left(1-ACC\left(x\right)\right)+\lambda \frac{k}{N}+\beta corr\left(x\right)$$

where *ACC*(**x**) is the accuracy rate obtained by the classifier on the test set (the percentage of well-classified patterns with regards to the total patterns analysed);  N is the total number of extracted features; and finally, *corr*(**x**) defines the correlation among the features and the target class, with the aim of avoiding the redundancy in the feature vector (equation 2). The parameters *λ *and *β *can take values in the interval (0, 1) and show how influential are the terms *minimisation of the number of genes *and *mutual information *in the fitness function. Further information is provided in the results section.

### C-MANTEC algorithm

C-MANTEC (Competitive Majority Network Trained by Error Correction) [17] is a novel neural network constructive algorithm that utilises competition between neurons and a modified perceptron learning rule to build compact architectures with good prediction capabilities. The novelty of C-MANTEC is that the neurons compete for learning the new incoming data, and this process permits the creation of very compact neural architectures. At the single neuronal level, the algorithm uses the thermal perceptron rule, introduced by Marcus Frean in 1992 [25], that improves the convergence of the standard perceptron for non-linearly separable problems. C-MANTEC, as a CNN algorithm [26,27], has in addition the advantage of generating online the topology of the network by adding new neurons during the training phase, resulting in faster training times and more compact architectures. Its network topology consists of a single hidden layer of thermal perceptrons that maps the information to an output neuron that uses a majority function.

The C-MANTEC algorithm has 3 parameters to be set at the time of starting the learning procedure. Several experiments have shown that the algorithm is very robust against changes of the parameter values and thus C-MANTEC operates fairly well in a wide range of values. The three parameters of the algorithm to be set are: (i) *Imax *as maximum number of iterations allowed for each neuron present in the hidden layer per learning cycle, (ii) *gfac *a growing factor that determines when to stop a learning cycle and include a new neuron in the hidden layer, and (iii) Phi (*φ*) that determines in which case an input example is considered as noise and removed from the training dataset according to Eq. 4:

(4) $$\forall X\in \left\{X_{1},...,X_{N}\right\},\;\text{delete}\left(\text{X}\right)|\text{NTL}\geq \left(\mu +\varphi \sigma \right)$$

where *X *represents a given pattern among the *N *patterns of the dataset, *NTL *is the number of times that pattern *X *has been learnt on the current learning cycle, and the pair {*µ,σ*} corresponds to the mean and variance of the normal distribution that represents the number of times that each pattern of the dataset has been learnt during the learning cycle. Thus, Eq. 4 specifies that if a given pattern (*X*) has been tried to be learnt by the network a number of times larger than *φ *standard deviations above the mean for the population it should be removed from the training set.

### Experimental results

In this section, six free-public cancer datasets (http://datam.i2r.a-star.edu.sg/datasets/krbd/index.html) have been used to test the proposed methodology. The main characteristics (# genes, # samples, and class distribution) for each dataset is shown in Table 1. A comparison between the two analyzed frameworks is conducted, where for each methodology six classification techniques are applied, namely: LDA, SVM, NaiveBayes, C-MANTEC, kNN and MLP.

**Table 1:** Cancer datasets

*Dataset*	*#Genes*	*Samples*	*Class 0 (**normal**)*	*Class 1 (**cancer**)*	*Data Proportion*
**Leukemia**	7129	72	25	47	0.347
**Lung**	12533	181	150	31	0.829
**Colon**	2000	62	22	40	0.355
**Breast**	24481	78	33	44	0.423
**Ovarian**	15154	253	91	162	0.360
**Prostate**	12600	102	50	52	0.490

Main characteristics of the six cancer datasets analysed.

Before applying the methodology based on genetic algorithms, it is necessary to estimate the parameters *α *and *β *associated with the fitness function and referred in a previous section. This estimation is carry out for all the cancer datasets, although only the information related to the *Lung *and *P rostate *datasets are shown by the sake of simplicity. Different combinations of the *λ *and *β *parameters together with the accuracy results on average and number of selected genes are shown in Table 2. The differences in the accuracy rates for each parameter combination are not statistically significant, which implies that, for these cancer datasets, any combination of parameters can be chosen. Specifically, the combinations *α *= 0.4, *β *= 0.25 and *α *= 0.1, *β *= 0.25 (Table 2, in italic), lead to the obtention of the largest success rate, taking into account that when *α *is reduced (*α *= 0.1) the number of genes in the solution is a little higher (12.78 in *P rostate *and 4.73 in *Lung*) than when we try to minimise the solution with more emphasis (*α *= 0.4, 9.32 genes in *P rostate *and 4.25 in *Lung*, on average).

**Table 2:** Parameters estimation for GA

Prostate dataset				Lung dataset			
*α*	*β*	*Accuracy*	*#Genes*	*α*	*β*	*Accuracy*	*#Genes*
0.8	0.6	0.9838*±*0.0097	2.67*±*1.19	0.8	0.6	0.9730*±*0.0107	8.65*±*2.82
0.8	0.4	0.9899*±*0.0072	3.30*±*1.02	0.8	0.4	0.9748*±*0.0093	7.28*±*1.20
0.8	0.25	0.9914*±*0.0054	3.52*±*0.91	0.8	0.25	0.9801*±*0.0106	9.85*±*3.12
0.4	0.6	0.9827*±*0.0086	2.56*±*1.01	0.4	0.6	0.9743*±*0.0103	8.80*±*3.18
0.4	0.4	0.9912*±*0.0069	3.75*±*1.44	0.4	0.4	0.9763*±*0.0094	9.55*±*1.08
*0.4*	*0.25*	*0.9938 ± 0.0061*	*4.25 ± 1.95*	*0.4*	*0.25*	*0.9849 ± 0.0089*	*9.32 ± 1.64*
0.1	0.6	0.9837 ± 0.0104	3.04 ± 1.71	0.1	0.6	0.9770 ± 0.0095	7.83 ± 2.06
0.1	0.4	0.9895 ± 0.0065	2.88 ± 0.70	0.1	0.4	0.9763 ± 0.0118	9.63 ± 2.53
*0.1*	*0.25*	*0.9966 ± 0.0041*	*4.73 ± 2.10*	*0.1*	*0.25*	*0.9854 ± 0.0101*	*12.78 ± 1.61*

Parameter estimation for the *α *and *β *parameters of the fitness function of the GA for the *Lung *and *Prostate *datasets.

**Table 3:** Parameters settings

Algorithm	Test Parameters
LDA	No parameters
SVM	Kernel type, *t*= {linear, polynomial, radial base function, sigmoid} Cost, *C *= {1, 3, 5, 7, 9, 10, 12, 15} Degree, *d *= {1, 2, 3, 4, 5} Gamma, *g *= {0.001, 0.005, 0.1, 0.15, 0.2, 0.4, 0.6, 0.8, 1, 2, 3, 5} Coef0, *r*= {0, 1, 2}
NaiveBayes	Kernel density, *K *= {0, 1} Supervised discretization, *D *= {0, 1}
C-MANTEC	Max. Iterations, *I_max _*= {1000, 10000, 100000} GFac, *g_fac _*= {0.01, 0.05, 0.1, 0.2, 0.25, 0.3} Phi, *φ *= {1, 1.5, 2, 2.5, 3, 3.5, 4, 4.5, 5, 5.5, 6}
kNN	Neighbours, *k *= {1, 2, 3, 4, 5, 6, 7, 8, 9, 10} Distance type, *d*= {Euclidean, chi-squared, cosine-similarity}
MLP	Hidden neurons, *N Hidden *= {2, 3, 4, 5, 6} Alpha, *α *= {0.05, 0.1, 0.2, 0.3, 0.5} Number cycles, *N Cycles *= {10, 25, 50}

Parameter settings tested during evaluation of the classification algorithms. The combination of all the values of the parameters generate a set of configurations for each method.

Table 3 shows the set of parameters that have to be set for each classifier, together with the different values that have been tested in this paper. For each classifier, a holdout validation strategy is used by dividing the entire dataset on a 60 − 40% proportion; the first set to train the model and the second to obtain the accuracy in the prediction of cancer outcome. The training-testing procedure is repeated 50 times randomly varying the training and testing set to avoid a biased evaluation, permitting also to analyse the dispersion of the results.

**Figure 1:**
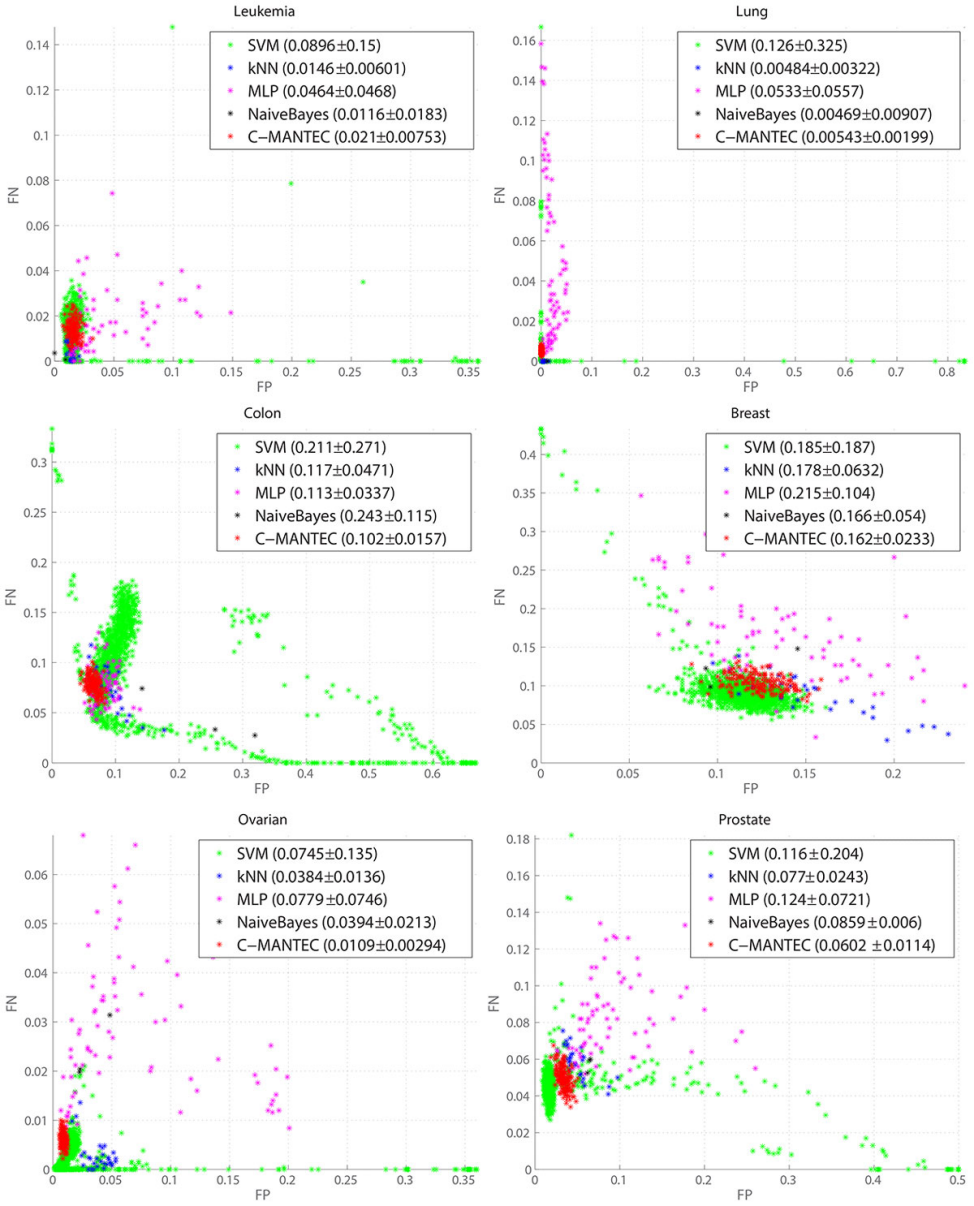
**Quantitative measures**. False Positives (FP) and False Negatives (FN) ratios after applying each method to the test sequences with all the parameter configurations. Each coloured point '*' is considered as a different configuration for the indicated method. The closer the points are to the origin, the better the segmentation. Additionally, the method is less sensible to a parameters' change if the cloud of points is more compact (see the text for more details). The datasets are different and so the scales are.

A thorough analysis of the parameter setting is presented in Figure 1, where its influence for the different algorithms is evaluated in the variability of the classification accuracy. The horizontal axis corresponds to the average percentage across the 50 samples considered of the false positives (*FP*) of the data, while the vertical axis is associated with the false negatives values (*FN*). Each point of the plot represents the *FP *and *FN *values of a generated configuration with a given parameter setting. The closer the points are to the origin, the better the classification accuracy, with optimum performance occurring for *FN *= *FP *= 0 (a perfect match between the output of the algorithm and the observed outcome of the dataset). All points are located always below the contradiagonal of the plot (*FN *+ *FP *= 1) as it is verified that *FN *+ *FP ≤ *1.

The variability observed for each classifier depends largely on the analysed dataset, but with the robustness of each of the method having also a strong influence, as more robust methods yield to more compact configuration clouds of points (a compact configuration cloud means that the results do not vary significantly after a change in the classifier parameters). Thus, the compactness can be defined as the standard deviation of the accuracy measures. As shown in Figure 1, the compactness for kNN, SVM and MLP methods is rather poor in general, while the C-MANTEC approach leads to configurations that are very close together, indicating clearly that the performance of this method is not very sensitive to the parameter selection. Additionally, C-MANTEC lead to the lowest values for the distance of the mean of the configuration values (*FP *and *FN*) to the origin, confirming the robustness in the parameter setting (the LDA classifier does not have parameters to be set and thus it is not represented in the graph). In order to quantify the distribution of the prediction accuracy observed for the several configuration analysed, the legend for each classifier shows the distance to the plot origin plus/minus the standard deviation $\left(\sqrt{{\left(FP\right)}^{2}+{\left(FN\right)}^{2}}\pm \text{std-dev}\right)$. For example, for the *Ovarian, Colon *and *Prostate *datasets, the distance to the origin for the mean value observed for the C-MANTEC algorithm is significantly lower than for the rest of alternatives (0.0109, 0.102 and 0.0602, respectively).

Comparison results between the two frameworks are shown in Table 4, where the best parameter configuration for each classification model is selected to perform the evaluation over the six datasets. In both frameworks, the accuracy rates for the *Leukemia, Lung *and *Ovarian *datasets are close to 100% regardless of the classifier applied, suggesting a low data complexity (in prediction terms). The complexity the *Breast, Colon *and *Prostate *seems higher, permitting to observe clear differences between the two approaches. The prediction accuracy obtained with the GA methodology was in almost all cases higher that the obtained within the SFS approach. Additionally, the robustness of the selected features is considerably higher in the GA (lower standard deviation values), fact that can be partially attributed to the larger set of genes selected. Regarding the computational complexity of both approaches, the SFS strategy involves approximately a number of comparisons of $n_{sel}\times \bar{\#genes}$ (*n_sel_*: number of pre-selected features, $\bar{\#genes}$: mean number of genes selected), while the GA approach utilises a maximum of 20.000 profile comparisons regardless of the dataset (length of the chromosome (100) *× *number of generations (200)). For example, for the *Prostate *dataset in the SFS approach, approximately 3000 comparison are needed in the present study since *n_sel _*≈ 600, $\bar{\#genes}=5$, unlike the genetic proposal which requires a greater number of combinations. However, if the number of pre-selected genes increases, the SFS method begins to loose its efficiency and may be intractable when handling thousands of genes.

**Table 4:** Performance comparison of classification techniques

			GA		SFS	
	***Classifier***	***Parameters***	***mean ± std***	***#genes***	***mean ± std***	***#genes***

**Leukemia**	LDA	-	99.959 ± 0.07	12	97.609 ± 2.86	2
	SVM	{polynomial,15,1,0.6,0}	99.982 ± 0.06	8	**99.918 ± 0.52**	4
	NaiveBayes	{1,0}	99.974 ± 0.03	12	98.060 ± 2.19	3
	C-MANTEC	{1000,0.01,4.5}	99.038 ± 0.25	7	98.837 ± 2.46	3
	kNN	{1,Euclidean}	**99.994 ± 0.02**	10	99.844 ± 0.77	5
	MLP	{3,0.5,50}	99.944 ± 0.05	5	95.784 ± 3.38	2
**Lung**	LDA	-	99.971 ± 0.03	5	99.057 ± 1.00	3
	SVM	{linear,10,-,-,-}	**100 ± 0**	11	99.828 ± 0.70	3
	NaiveBayes	{1,0}	99.998 ± 0.01	4	**99.991 ± 0.07**	3
	C-MANTEC	{100000,0.25,2}	99.678 ± 0.08	6	99.673 ± 0.94	2
	kNN	{1,Euclidean}	99.969 ± 0.02	4	99.969 ± 0.22	4
	MLP	{4,0.1,50}	99.996 ± 0.01	4	99.778 ± 0.79	2
**Colon**	LDA	-	98.676 ± 0.35	11	87.179 ± 6.15	2
	SVM	{polynomial,1,1,0.4,2}	89.917 ± 1.26	20	91.738 ± 5.21	5
	NaiveBayes	{0,1}	90.583 ± 0.49	15	89.076 ± 7.79	4
	C-MANTEC	{10000,0.01,1}	94.315 ± 0.48	11	87.593 ± 6.69	2
	kNN	{3,cosine-similarity}	95.060 ± 0.38	19	**93.577 ± 4.43**	6
	MLP	{5,0.3,50}	**99.026 ± 0.30**	12	88.733 ± 5.51	2
**Breast**	LDA	-	99.788 ± 0.12	15	74.169 ± 6.52	1
	SVM	{polynomial,7,2,0.001,2}	99.744 ± 0.14	31	**81.029 ± 5.80**	3
	NaiveBayes	{0,0}	97.759 ± 0.23	27	73.499 ± 6.34	1
	C-MANTEC	{10000,0.01,1.5}	97.342 ± 0.39	23	76.645 ± 6.53	1
	kNN	{3,Euclidean}	97.485 ± 0.30	34	80.975 ± 6.37	2
	MLP	{4,0.3,50}	**99.828 ± 0.09**	18	79.191 ± 6.43	2
**Ovarian**	LDA	-	99.980 ± 0.01	4	**100 ± 0**	3
	SVM	{polynomial,9,1,0.2,0}	**100 ± 0**	4	99.978 ± 0.13	4
	NaiveBayes	{1,0}	99.951 ± 0.03	5	99.980 ± 0.13	4
	C-MANTEC	{1000,0.3,1.5}	99.844 ± 0.05	4	99.659 ± 0.75	3
	kNN	{1,Euclidean}	99.984 ± 0.01	4	99.982 ± 0.11	3
	MLP	{5,0.3,50}	99.999 ± 0	3	**100 ± 0**	3
**Prostate**	LDA	-	99.720 ± 0.12	9	95.677 ± 2.81	4
	SVM	{polynomial,5,1,3,1}	99.428 ± 0.31	20	**98.622 ± 1.79**	5
	NaiveBayes	{0,0}	98.817 ± 0.16	14	98.331 ± 2.13	7
	C-MANTEC	{1000,0.25,4}	98.681 ± 0.24	8	95.351 ± 3.40	4
	kNN	{3,cosine-similarity}	99.633 ± 0.11	20	97.146 ± 2.28	6
	MLP	{3,0.5,50}	**99.996 ± 0.02**	12	96.921 ± 2.37	4

Performance comparison among the two different feature selection frameworks used (GA and SFS) and the six classifiers analyzed (LDA, SVM, NaiveBayes, C-MANTEC, kNN and MLP) for each cancer microarray dataset. The results correspond to the best simulation for each dataset, showing the accuracy for method in the format of *mean ± standard deviation *and the number of selected genes.

Table 5 shows average results across all six datasets for the both frameworks used, noting that C-MANTEC lead to competitive classification performance with a reduced number of genes.

**Table 5:** Performance comparison of feature selection frameworks

	GA		SFS	
**Classifier**	***mean ± std***	***#genes***	***mean ± std***	***#genes***

LDA	99.682 ± 0.12	9.33	92.282 ± 3.22	2.5
SVM	99.082 ± 0.25	15.67	95.185 ± 2.36	4
NaiveBayes	97.847 ± 0.16	12.83	93.156 ± 3.11	3.67
C-MANTEC	98.150 ± 0.25	9.83	92.960 ± 3.46	2.5
kNN	98.688 ± 0.14	15.17	95.249 ± 2.36	4.33
MLP	99.798 ± 0.08	9	93.401 ± 3.08	2.5

Average performance comparison among two different feature selection frameworks (GA and SFS) and six classifiers (LDA, SVM, NaiveBayes, C-MANTEC, kNN and MLP) over all dataset.

Further, we analyzed the differences between classifiers for the SFS and GA feature selection procedures used and for the six datasets, showing the results in Table 6. The corresponding p-value obtained after applying a Friedman's test is indicated in the third column [28]. In case this p-value is lower than 0.05, the lowest performant classifier is taken as a control group and the last column of the table lists the classifiers that lead to statistically significant results (from the lowest to the highest difference); otherwise, non statistically significant results are reached (represented with a "-" on the table).

**Table 6:** Differences between classifiers.

FS procedure	Dataset	p-value	Control	Statistically different classifiers
SFS	Leukemia	*< e*^−16^	LDA	SVM
	Lung	*< e*^−16^	LDA	kNN, NB
	Colon	*< e*^−16^	LDA	SVM, kNN
	Breast	*< e*^−16^	NB	kNN, SVM
	Ovarian	*< e*^−16^	CM	LDA, NN
	Prostate	*< e*^−16^	CM	NB, SVM
GA	Leukemia	*< e*^−16^	CM	NB, NN, LDA, SVM, kNN
	Lung	*< e*^−16^	CM	SVM, NB, NN
	Colon	*< e*^−16^	SVM	LDA, NN
	Breast	*< e*^−16^	SVM	NN, LDA
	Ovarian	*< e*^−16^	CM	SVM, NN
	Prostate	*< e*^−16^	CM	NN, LDA

Differences between classifiers for the two feature selection (FS) procedures used (first column). The lowest performance classifier is taken as control group and the last column of the table lists the classifiers that lead to statistically significant results (corresponding p-value indicated in the third column).

Table 7 shows a similar comparative analysis but among the SFS and GA feature selection procedures when a common classifier is used (first column of the table).

**Table 7:** Differences between feature selection algorithms

Classifier	Dataset	p-value	Control	Statistically different FS procedures
LDA	Leukemia	1.54*e*^−12^	SFS	GA
	Lung	1.54*e*^−12^	SFS	GA
	Colon	1.54*e*^−12^	SFS	GA
	Breast	1.54*e*^−12^	SFS	GA
	Ovarian	3.28*e*^−11^	GA	SFS
	Prostate	1.54*e*^−12^	SFS	GA
SVM	Leukemia	3.65*e*^−5^	SFS	GA
	Lung	1.54*e*^−12^	SFS	GA
	Colon	2.86*e*^−9^	GA	SFS
	Breast	1.54*e*^−12^	SFS	GA
	Ovarian	9.13*e*−11	SFS	GA
	Prostate	1.54*e*−12	SFS	GA
NB	Leukemia	4.71*e*−9	SFS	GA
	Lung	1.54*e*^−12^	SFS	GA
	Colon	1.54*e*^−12^	SFS	GA
	Breast	1.54*e*^−12^	SFS	GA
	Ovarian	0.157	-	-
	Prostate	1.54*e*^−12^	SFS	GA
CM	Leukemia	4.71*e*^−9^	SFS	GA
	Lung	1.54*e*^−12^	SFS	GA
	Colon	1.54*e*^−12^	SFS	GA
	Breast	1.54*e*^−12^	SFS	GA
	Ovarian	0.157	-	-
	Prostate	1.54*e*^−12^	SFS	GA
kNN	Leukemia	1.54*e*^−12^	SFS	GA
	Lung	0.0897	-	-
	Colon	1.54*e*^−12^	SFS	GA
	Breast	1.54*e*^−12^	SFS	GA
	Ovarian	0.6547	-	-
	Prostate	1.54*e*^−12^	SFS	GA
NN	Leukemia	4.71*e*^−9^	SFS	GA
	Lung	1.54*e*^−12^	SFS	GA
	Colon	1.54*e*^−12^	SFS	GA
	Breast	1.54*e*^−12^	SFS	GA
	Ovarian	0.157	-	-
	Prostate	1.54*e*^−12^	SFS	GA

Differences between SFS and GA feature selection algorithms for the six different classification methods used (first column). The lowest performant FS procedure is taken as control group (fourth column) while the last column of the table lists the procedures that lead to statistically significant results (corresponding p-value indicated in the third column)

### Biological analysis

**Figure 2:**
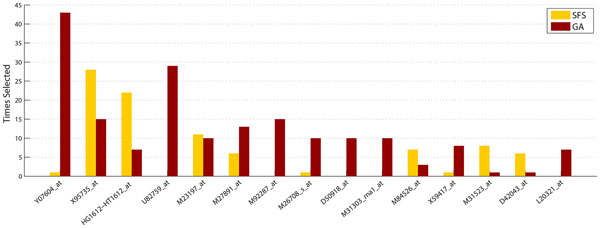
**Frequency selection of genes for Leukemia, Lung, Colon and Breast databases**. The ten most selected features for the analysed datasets. Frequency selection is represented by an horizontal bar, divided according to the six classifiers used in the analysis: LDA, SVM, C-MANTEC, kNN, NaiveBayes and MLP. The index, gene symbol and probe set ID of each gene is shown in columns one to three.

**Figure 3:**
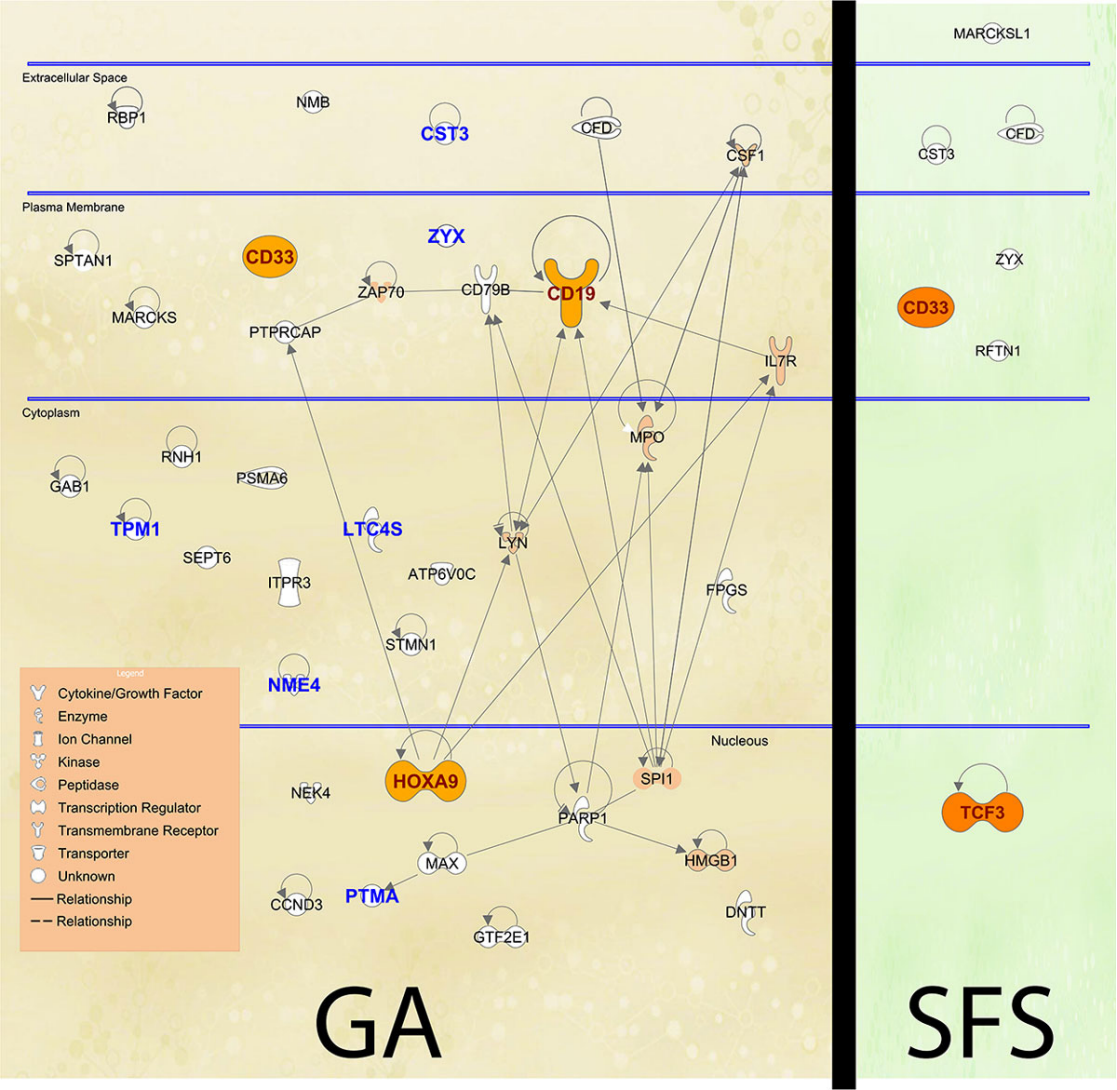
**Frequency selection of genes for Ovarian and Prostate databases**. The ten most selected features for the analysed datasets. The structure of this figure is the same than Figure 2.

Figures 2 and 3 present the ten most selected genes for each of the six datasets considered, where each dataset is represented in a row of the table. The first three columns show information about the gene, such as the internal index (ID), the gene symbol (name of the gene) and the probe set ID, which is related to the chip where the dataset has been extracted (e.g., Affymetrix). The bar graph of the last column splits the frequency of selection (fourth column) of each feature according to the GA-LDA, GA-SVM, GA-CMANTEC, GA-kNN, GA-NaiveBayes and GA-MLP strategies. Most of the gene symbols have been found from their probe set ID by using tools as IPA (Ingenuity^® ^Systems, http://www.ingenuity.com) or NCBI (http://www.ncbi.nlm.nih.gov/gene/), although it has not been possible for the *Ovarian *dataset (first row of Figure 3) because there is no reference of the chip from which the data have been extracted.

A higher frequency of selection might imply a higher relevance of the gene in the prognosis of the disease. Those genes that are selected with similar frequency for all classifiers are considered independent with respect to the classification method. For instance, in the *Prostate *dataset (second row of Figure 3), the MAF gene is more significant than the JUNB gene, since it has been selected more times and all the classifiers selects it with the same frequency. Thus, *NaiveBayes *barely takes into account the JUNB gene whereas for MLP classifier it is one of the main genes.

Not only are we interested in getting good results in prognosis prediction but also in examining whether the selected genes provide biological information related to the disease studied. Therefore, if the proposed models provide this consistency between the computational and biological field, the results would be more confident and the selected genes would be more reliable from a clinical perspective, in order to their implementation in microchips and treatment in real patients. We can see that this statement is true in the proposed model using genetic algorithms.

In the case of the *Prostate *dataset is possible to find references in the literature where the genes **MAF**, which encodes a protein related to DNA-binding (most frequent gene, 99.67%) [29], **SERPINB5**, a serpin peptidase inhibitor (second most frequent, 58%) [30], **HPN**, officially named hepsin which encodes a type II transmembrane serine protease (fourth most frequent, 50%) [31] and **GSTP1**, belonging to the family of Glutathione S-transferases (GSTs) enzymes (sixth most frequent, 36.33%) [32] are biologically related to the absence or presence of prostate cancer. This supports the idea that our computational approach is robust and consistent with the results obtained in biological studies.

For the *Breast *dataset, several of the most selected genes among which are **UBC **[33,34], **ZNF222 **[35] and **EWSR1 **[36], are biologically associated with breast cancer. The same happen for the *Leukemia *disease, where the enforced expression of the **CD19 **molecule (fifth selected, 19%) can reduce the proliferation of the malignant plasma cells [37]; the gene homeobox A9 (**HOXA9**, second selected, 33%) influences hematopoietic progenitors and acute leukemias [38]; and the **CD33 **molecule (seventh selected, 17.33%) has been shown to sharply inhibit the in vitro proliferation of both normal myeloid cells and chronic myeloid leukemias [39].

From a computational point of view, Table 8 shows the best selected genes obtained by the genetic approach which also have been extracted in several related papers (last column of the table) for the particular case of the *Leukemia *dataset. It should be noted that the applied methodology is different from one paper to another. For instance, five of the ten genes are also reported in the list of the 50 most important genes (selected from 7129) suggested in [40].

**Table 8:** Selected genes for the Leukemia dataset

ID	Probe Set ID	Gene Description	References
4951	*Y07604_at*	NME/NM23 nucleoside diphosphate kinase 4	[41-43]
3847	*U82759_at*	Homeo box A9	[40,44,43]
6169	*M13690_s_at*	C1NH Complement component 1 inhibitor	[43,45]
6184	*M26708_s_at*	PTMA Prothymosin alpha	[45]
6225	*M84371_rna1_s_at*	CD19 Molecule	[46]
1882	*M27891_at*	CST3 Cystatin C	[40,41,43,44]
1834	*M23197_at*	CD33 antigen	[40,44,46]
4847	*X95735_at*	Zyxin	[40,41,44,46]
3320	*U50136_rna1_at*	LTC4 synthase	[40,43,44,46]
5094	*Z24727_at*	TPM1 Tropomyosin alpha chain	[47]

The best selected genes ranked with the GA approach for the *Leukemia *dataset which also appear in other studies in the literature.

**Figure 4:**
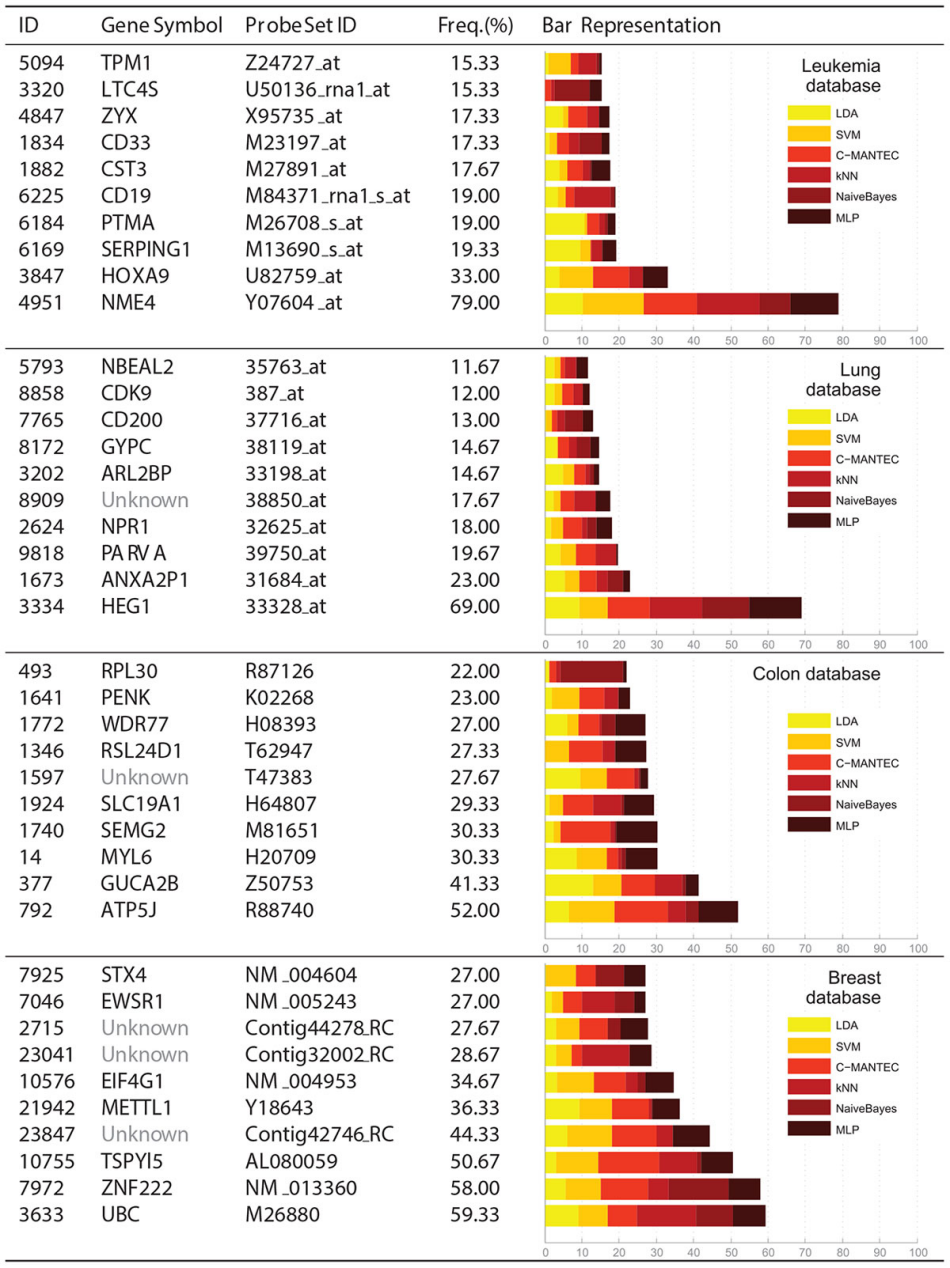
**Comparison of the most frequently selected genes**. Comparison of the most frequently selected genes (in 50 independent executions) by the GA and SFS strategy in Leukemia dataset, with independence of the classifier used.

Focusing on the *Leukemia *dataset (one of the most studied dataset in the literature), and as a biological analysis of the features selected, Figure 4 displays a comparison between the most selected genes, after 50 independent executions and with independence of the classifier used, for both GA and SFS selection procedures. Moreover, the IPA tool is used in order to explore the functional involvement of each gene set obtained by GA and SFS in the studied disease. In concrete, three of the fifteen most frequently genes are highlighted in bold on the *x*-axis in Figure 4 as founded genes in the IPA database with biological relevance on the Leukemia cancer disease.

**Figure 5:**
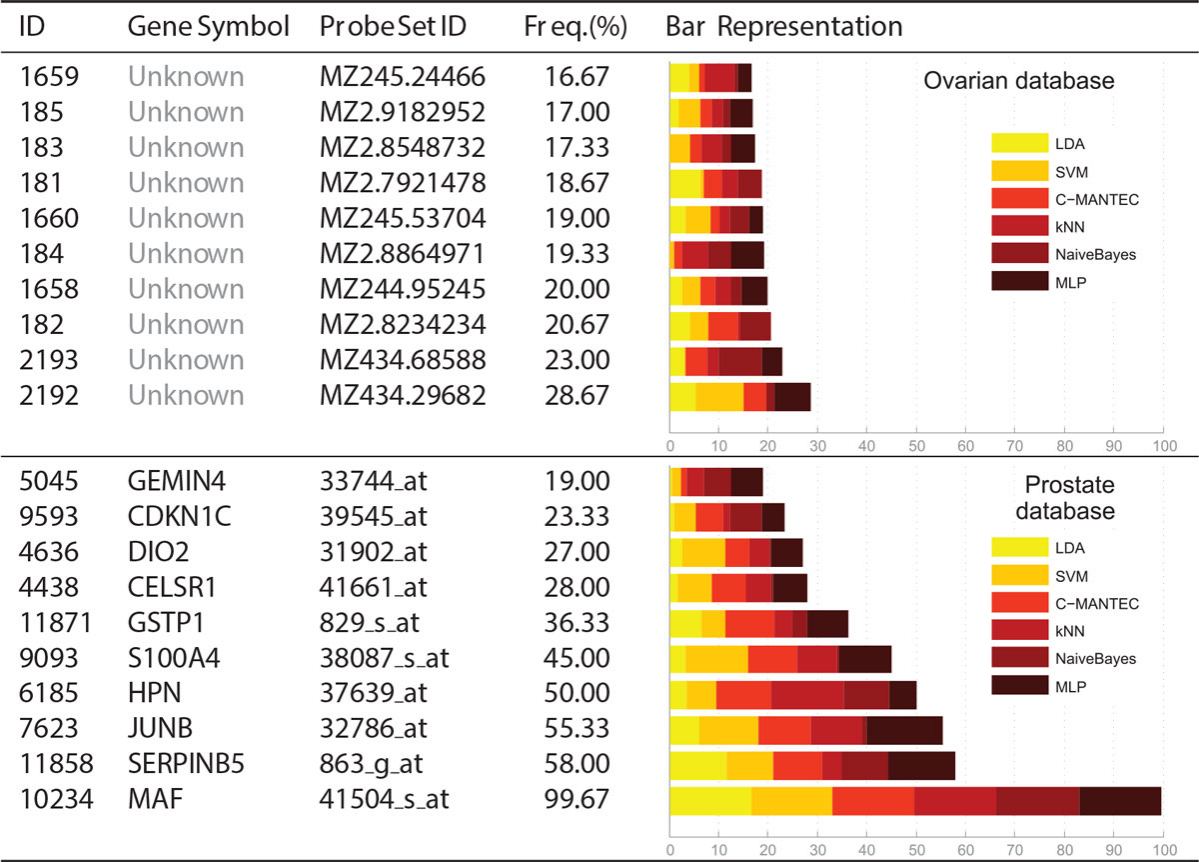
**Biological analysis for Leukemia dataset**. Biological analysis of the resuls obtained by GA-CMANTEC and SFS-CMANTEC strategy for the ***Leukemia ***dataset using the IPA tool.

A deeper biological analysis is performed using the IPA tool for the GA-CMANTEC strategy considering the *Leukemia *dataset. Figure 5 shows those genes that are selected at least a 5% of the times both with GA-CMANTEC or SFS-CMANTEC strategy after 50 independent executions. The names shown on this figure correspond to the symbol of each gene according to Figure 2. It is important to highlight the difference on the number of genes selected through the GA and SFS strategy due to the casuistic of each algorithm. Additionally, on the left side are represented in bold nine of the ten most frequently selected genes with independence of the classifier used. Moreover, using C-MANTEC as classifier allow to obtain these nine most selected genes. Finally, filled in genes represent those genes that have demonstrate biological relevance on the Leukemia disease. In this sense, the GA-CMANTEC strategy presents 10 out of 37 genes as a result while the SFS-CMANTEC strategy presents 2 out of 7. Although these results are similar in proportion, the GA-CMANTEC strategy could be considered more explicative from a biological point view with no detriment on the classification performance. Furthermore, the connections among the selected genes (represented by links in Figure 5), which are more numerous in the GA approach, suggest as well a significant relationship with the occurrence of the disease.

## Conclusions

In this work, a new methodology approach combining genetic algorithm with constructive neural networks has been proposed in order to predict cancer outcome. For six free-public cancer datasets, we compared under GA and SFS frameworks the prediction accuracy of the C-MANTEC algorithm against the following five standard classifiers: LDA, SVM, NaiveBayes, kNN or MLP.

On average, the strategy based on the GA approach leads to better prediction rates, observing that these results are independent of the classifier used, noting also that prediction results under the GA framework show lower variability, and thus can be considered as more robust. On the other hand, it should be noted that the SFS approach is less computationally intensive, involving in the present study approximately seven times less gene comparisons, and also leading to a group of selected genes much smaller than those selected under the GA approach. Nevertheless, when complex datasets are studied like *Breast *or *Colon*, cancer prognosis results are quite poor when using the SFS approach, presumably since the search in the state space is much more restrictive. Additionally, an analysis done using the IPA methodology suggests that the biological relevance of the genes selected under the GA framework is higher than the observed using the SFS approach, as indicated by the reference frequency in the literature and also regarding the relationship between them (even if this effect might be due to the size of both selected sets).

Regarding the comparison between the different classifiers implemented, standard feedforward neural networks (MLP), LDA and SVM lead to similar and best results while C-MANTEC and kNN followed closely but with a bit lower accuracy. C-MANTEC, MLP and LDA permitted to obtain a more reduced set of genes in comparison to SVM, NB and kNN. Further, C-MANTEC resulted in the most robust classifier in terms of changes in the parameter settings, a relevant feature for its use in wrapper feature selection methods (as it will reduce execution times related to parameter tuning). Additionally, we are considering the use of a ensemble of all these classifiers as a further work, in order to obtain a greater consensus on the classification result, which could lead to greater robustness and accuracy of the resulting model.

## Competing interests

The authors declare that they have no competing interests.

## Authors' contributions

RML, DU and JMJ contributed to the conception and design of the study. RML, DU and JLS contributed to write the programming code. RML, DU, JMJ and LF contributed to the analysis and interpretation of the data, and RML, DU, LF and JMJ contributed to the drafting of the manuscript. All authors approved the manuscript.

## Declarations

Publication funding for this article has come from grants TIN2010-16556 (MICINN-Spain) and TIC-4026/2008 (Consejería de Innovación, Ciencia y Empresa. Junta de Andalucía), both including FEDER funds. Additionally, the authors acknowledge support through these grants.

This article has been published as part of *Theoretical Biology and Medical Modelling *Volume 11 Supplement 1, 2014: Selected articles from the 1st International Work-Conference on Bioinformatics and Biomedical Engineering-IWBBIO 2013. The full contents of the supplement are available online at http://www.tbiomed.com/supplements/11/S1.
